# Age-Related Differential Effects of School-Based Sitting and Movement Meditation on Creativity and Spatial Cognition: A Pilot Study

**DOI:** 10.3390/children8070583

**Published:** 2021-07-08

**Authors:** Fabio Marson, Antonio De Fano, Michele Pellegrino, Caterina Pesce, Joseph Glicksohn, Tal Dotan Ben-Soussan

**Affiliations:** 1 Research Institute for Neuroscience, Education and Didactics, Patrizio Paoletti Foundation, 06081 Assisi, Italy; f.marson@fondazionepatriziopaoletti.org (F.M.); m.pellegrino@fondazionepatriziopaoletti.org (M.P.); 2 Neuroimaging Laboratory, Department of Physiology and Pharmacology, Sapienza University, 00185 Rome, Italy; 3 Neuroscience and Imaging, Behavioral Imaging and Neural Dynamics (BIND) Center, Department of Neuroscience, Imaging and Clinical Sciences, University “G. d’Annunzio” of Chieti-Pescara, 66100 Chieti, Italy; antonio.defano@unich.it; 4 Laboratory of Experimental Neuropsychology, Department of Psychology, Sapienza University, 00185 Rome, Italy; 5 Department of Movement, Human and Health Sciences, University of Rome Foro Italico, 00135 Rome, Italy; caterina.pesce@uniroma4.it; 6 Department of Criminology, Bar-Ilan University, Ramat Gan 5290002, Israel; gliksoj@biu.ac.il; 7 The Leslie and Susan Gonda (Goldschmied) Multidisciplinary Brain Research Center, Bar-Ilan University, Ramat Gan 5290002, Israel

**Keywords:** school-based, movement, meditation, spatial cognition, creativity, cognitive flexibility

## Abstract

Psychophysical well-being can be supported during development by the integration of extra-curricular activities in scholastic settings. These activities can be implemented in different forms, ranging from physical activities to sitting meditation practices. Considering that both such activities are thought to affect children’s psychophysical development, a movement-based meditation that combines the two approaches−in the form of a short daily activity−could represent a powerful tool to promote healthy physical and mental development. Consequently, the current pilot study aimed to examine the effect of short daily school-based sitting and movement meditation trainings on creativity and spatial cognition. Utilizing a crossover design, we evaluated their feasibility and efficacy at different ages among children (*n* = 50) in 5th to 8th grade. We observed that 5 weeks of daily training in sitting and movement meditation techniques improved children’s cognition differently. Specifically, younger children showed greater creativity and better spatial cognition following the movement-based meditation, while older children showed greater enhancement in these areas following sitting meditation training. This suggests that training can affect children’s cognition differently depending on their developmental stage. We discuss these results within the framework of embodied and grounded cognition theories. Information on feasibility and age-related effect sizes derived from the current study paves the way for future well-powered larger-scale efficacy studies on different forms of school-based interventions to cognitive development promotion.

## 1. Introduction

Childhood is an important period characterized by great physical, social, cognitive, and emotional changes. This multifaceted development can be enhanced by the experiences and activities to which children are exposed. Indubitably, elementary and middle schools are environments that play a major role in providing opportunities to promote children’s positive developmental trajectory. As suggested by different studies, physical activity is particularly important to foster general psychophysical well-being, as well as improvements in cognitive and emotional functioning [1,2,3,4]. However, regular, mainstream scholastic programs generally do not appear to sufficiently support physical development [5], necessitating ad-hoc school-based interventions to properly support healthy child and adolescent development. In fact, two recent meta-analyses showed how such school-based physical activity (PA) interventions may have positive effects on various physical and non-physical domains, such as quality of life, self-efficacy, well-being, prosocial behaviors and enjoyment [6], reduced psychiatric symptoms, and enhanced resilience [7].

Interestingly, physical activity has been associated with increased cognitive flexibility [8,9] and better performance on tasks involving spatial processing [10]. Contextually, physical activity has also been associated with structural changes of brain regions underlying such cognitive functions [11,12,13]. In turn, cognitive flexibility is thought to play a role in promoting positive emotions, resilience, psychological well-being and high-order cognitive functions like creativity [14,15,16,17,18], while spatial cognition is thought to underlie reasoning [19] and a large variety of mental processes [20], including social interactions [21].

Cognitive flexibility and spatial cognition are two cognitive functions that were showed to be positively affected not only by PA-based interventions, but also by meditation-based interventions (e.g., [22,23,24,25]). Similar to PA, meditation is a practice that could be easily implemented in school settings and integrated with the rest of the traditional educational activities. Therefore, it is believed to be a suitable and effective mean to promote children’ general psychophysical well-being and foster cognitive-affective functioning [26,27]. Improvement in cognitive functioning following mindfulness-based interventions has even superseded by other positive psychological outcomes, such as coping and resilience [28].

Similarly to PA, meditation seems to share different basic mechanisms with creativity, suggesting a close interrelation between the practice of meditation and the ability to think creatively [29]. For example, both meditation and creativity have been associated with greater cognitive flexibility [30], increased short-term memory capacities [31,32,33] and changes in the regulation of information processing through modulation of attention over time [34,35,36].

Physical activity and meditative practices, respectively, could be implemented in educational contexts in a large variety of forms differing in duration, activity type, session length, and intensity [26,37]. Notwithstanding, this implementation can be complex. For example, one of the most widespread approaches is to engage children with activities lasting 30 to 60 min, making integration in an ecological school setting challenging. Moreover, the efficacy of meditation-based interventions seems to be positively associated with the number of daily sessions, in addition to the experience of the teacher/practitioner leading the meditation sessions [26], which presents another challenge for implementation in a school setting.

Among the various types of interventions based on physical activity explored in school settings, ‘active breaks’ and ‘brain breaks’ seem to be particularly feasible and efficacious interventions [38,39]. Active breaks are short (5 to 15 min) sessions of physical activity aimed to reduce sitting time, stimulate physical and cognitive activation, and promote on-task behaviors during school time [40]. Active breaks are associated with enhanced cognitive functions [41,42], especially when involving cognitively engaging movements [43,44,45]. Advantages of such intervention strategies, in addition to improving well-being, are that they are time sparing and require little space and cost. Considering the potential benefits of physical activity and meditative practices on children’s psychophysical, emotional, and cognitive well-being via enhanced cognitive flexibility and spatial processing, we suggest that trainings combining physical activity and meditation requiring low-intensity activity in short daily bouts would be easily implemented in an ecological scholastic context without interfering too much with regular didactic activities, providing a valuable and feasible support for healthy development. 

To this aim, we chose two short and low-intensity meditative practices which differ mainly on one key aspect, which is the execution of movement. Quadrato Motor Training (QMT) is a movement meditation practice that combines sensorimotor control, inhibitory control, and divided attention in order to foster bodily self-awareness and psychological well-being [46,47]. Previous research conducted in laboratory environments with adults have shown that QMT improves visuospatial abilities, creativity, neuroplasticity, and functional connectivity [48,49,50]. Such findings on the cognitive and neurophysiological benefits of QMT among adults, and one study that included also children [51], suggest that it could be a particularly valuable practice to promoting health and psychological wellbeing during development.

The training adopted for the current study included the aforementioned QMT as well as a short meditation practice called the One Minute Meditation (OMM; [52]). These two trainings crucially differ in how they engage participant experience. Specifically, QMT is designed to involve motor control and executive functions, such as divided attention and inhibition, in order to elicit the embodied experience of being in the here-and-now [46]; OMM is a structured sitting meditation in which the experience of being in the here-and-now is elicited without the execution of bodily movements [52].

Both creativity, spatial cognition and visuospatial recall were previously reported to improve following different types of meditation [24,30,53,54]. Yet the evidence regarding the connection between meditation and creativity is inconsistent, possibly dependent on the type of meditation. More specifically, focused attention meditation and open monitoring meditation may induce two different cognitive-control states that support state-compatible thinking styles, such as convergent and divergent thinking. So, while focused attention meditation may improve convergent thinking, divergent thinking can be significantly enhanced after open monitoring meditation [29]. While meditation has been generally categorized into these two types (in which focused attention meditation requires voluntary focusing of attention on a chosen object, and open monitoring meditation involves non-reactive monitoring of the content of experience from moment to moment), most meditative techniques lie somewhere on a continuum between the poles of these two methods [55]. For example, QMT includes both aspects, requiring both divided attention between body and external verbal commands, in parallel to waiting to the next command [56].

Furthermore, creativity may also depend on visuo-spatial abilities, and especially the ability to generate, maintain, and transform a visual image, in terms of response accuracy and response times [57]. Thus, with the aim of examining the differential effect of sitting and movement meditation, we selected two training types that had the same theoretical background and mainly differed in the degree of movement involved. The common theoretical background was that of the Sphere Model of Consciousness (SMC), which is a neurophenomenological geometric model focused on the subjective experience of the world derived from the awareness of the body in space and time [58,59]. According to the SMC, regulation of behavior through mindful training can expand spatial cognition. This, in turn, seems to be associated with creativity [57]. Especially, within the multidimensional–perceptual and imagery-construct of spatial ability, object and spatial imagery and visualization [60] may lie at the intersection of the ability necessary to mentally represent objects from different perspectives needed to perform the Divergent Thinking task successfully and the ability to represent the body in the space that underlies the space experience of the SMC-based sitting and movement meditation. Moreover, by purposefully practicing mindful training one can enhance attentional regulation and meta-observation [61,62,63], which in turn can enhance spatial cognition, ideational flexibility, and reduce cognitive rigidity [24,64,65], thus allowing a shift from the more habitual memory based narrative self to the minimal self which is embodied in the here and now.

The theoretical background of the SMC has its roots, among others, in three main lines of studies emphasizing the relationship between higher states of consciousness, spatial cognition and creativity, namely: (1) meditation, such as Open Monitoring Meditation, demonstrating that reduction of top-down control favors the emergence of divergent thinking [30,54]; (2) consciousness without content [66,67], and (3) the binary distinction between Minimal and Narrative Selves and their relation to meditative practices, especially to the embodied experience of here and now versus mind wandering [68].

Consequently, the current study aimed to explore the effects of short daily sitting and movement meditation trainings on creativity and spatial cognition in a school environment. It was developed as a pilot study with a crossover design to properly disentangle the effects of the two meditation types, and was conducted among children in 5th–8th grades to evaluate its feasibility and efficacy at different ages. This allowed us to obtain information on feasibility and age-related effect sizes that can inform well-powered future studies in school environments. The reporting, therefore, adheres to CONSORT guidelines for feasibility and pilot studies [69].

## 2. Materials and Methods

### 2.1. Sample Description

As a pilot study, no sample size calculation was performed. Four different classes from the same Montessori-oriented school were involved in this feasibility study. The classes involved were the 5th (*n* = 15; *F* = 4, *M* = 11), 6th (*n* = 15; *F* = 5, *M* = 10), 7th (*n* = 9; *F* = 4, *M* = 5), and 8th (*n* = 11; *F* = 5, *M* = 6) grades (total *N* = 50). The mean ages of children within their grades were 10, 11, 12 and 13 years old, respectively. To ensure ecological validity and avoid social exclusion for ethical reasons, all children, including those with Special Educational Needs, participated in the trainings and assessments together. However, to avoid any differential influence of atypical development on the primary outcome, children with Special Needs or any other type of diagnosis that could affect the intervention outcomes (*n* = 7) were excluded from the analysis. Before starting the intervention, informed parental consent was obtained.

### 2.2. Training Description

Children performed two types of meditative-based training named, respectively, Quadrato Motor Training (QMT) and One Minute Meditation (OMM).

**Figure 1:**
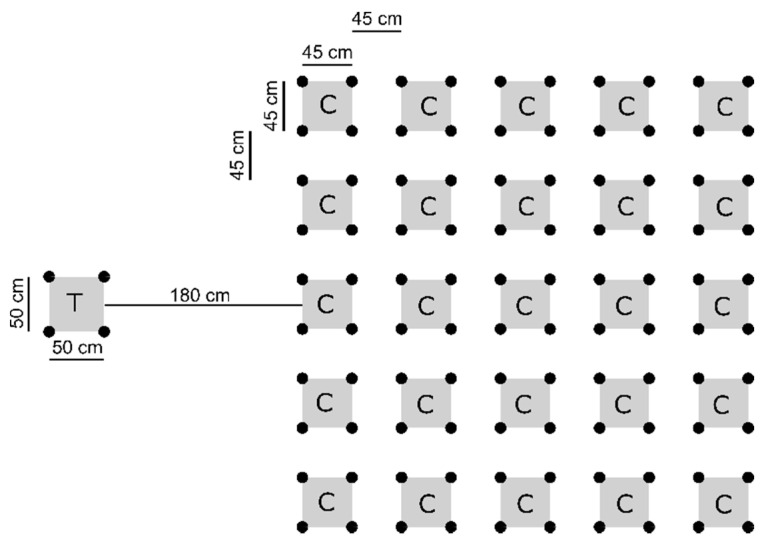
Disposition of squares for the Quadrato Motor Training (QMT) group training. The square reserved to the teacher is indicated with “T”, while the squares reserved to children are indicated with “C”.

QMT is a sensorimotor training that consists of performing forward, backward, lateral, and diagonal footsteps over the angles of a pre-defined square (in Italian, “Quadrato” means square) according to vocal instructions [46,47]. QMT aims to stimulate a sense of being in the here-and-now through activation of bodily self-awareness. The duration of this training was ~5 min. Each child had their own square in which they performed the training. All squares were arranged within the same experimental facility (i.e., school gym), allowing children to perform the training simultaneously (see Figure 1). Before each session, a schoolteacher accompanied the children from the classroom to the gym. Then, children were randomly assigned to a square. The square assigned to each child could change across sessions. A trained practitioner guided the QMT sessions, providing the children with vocal instructions and, when necessary, feedback concerning the correct execution of the given instruction.

OMM is a brief sitting meditation technique that consists of staying silent with one’s eyes closed while directing attention on interoceptive feelings (i.e., breathing) and envisioning positive beliefs and desires about the self [52]. OMM aims to promote self-awareness and the sense of being in the here-and-now by stimulating participants to envision the ‘best’ of themselves while focusing on bodily sensations. As in the case for QMT, each daily OMM session was performed in groups, guided by a trained practitioner; the duration of this training was ~5 min in total. Training duration includes the actual meditation (one minute) and few preliminary steps guiding the participants to get prepared for the meditation session by focusing on positive evaluation of the self, to enhance determination, and detachment from judgmental behaviors [52,59].

### 2.3. Experimental Design

This study adopted a crossover, repeated measures longitudinal design in which children received the two types of meditative training sequentially. During the first 10 weeks, the 5th and 7th grade classes performed the QMT, while the 6th and 8th classes performed the OMM. Allocation of classes to one training or another did not follow any specific rule, except that two consecutive classes could not do the same training in the same phase to balance training with developmental stage. Then, the classes switched their respective trainings and started the second 10-weeks training phase. A 4-week washout period corresponding to the length of the winter holiday break separated the two training phases. A washout is targeted to be long enough to rule out the effects of the first training phase [70]; however, to the best of our knowledge, there is currently no consensus on how long a washout period should last to erase any cognitive improvements from meditation-based trainings. Thus, aiming to maximize feasibility, we chose a washout period synchronized with the ecological school schedule context.

**Figure 2:**
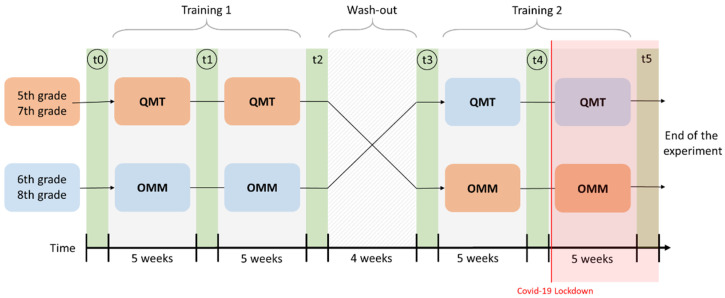
Original design with assessments (in green), training phases (in grey) and the washout. Encircled time-points represent the assessments considered for analyses. Highlighted in red is the part that could not be completed because of the COVID-19 lockdown in March 2020. QMT = Quadrato Motor Training; OMM = One Minute Meditation.

Cognitive assessments were administered before (T0 and T3), during the middle (i.e., 5 weeks; T1 and T4) and after (i.e., 10 weeks; T2 and T5) each training phase. Unfortunately, the study had to be interrupted at T4 due to the COVID-19 lockdown (Figure 2). However, the design can still be considered valid if the study must be truncated to three periods [71].

### 2.4. Test Description

Two paper-and-pencil tests were used for cognitive assessments: (1) the Hidden Figure Test (HFT) and (2) the Alternative Uses Test (AUT), measuring spatial cognition and creativity, respectively. Both tests were performed in groups in an ecological school context (i.e., in each school class).

Our choice of these tasks was guided by the theoretical approach and empirical (behavioral and neurophysiological) evidence from previous research in this domain with adults, indicating that QMT leads to an improvement in AUT performance—both flexibility and fluency [72] and HFT performance [73], as well as to modifications in brain structure and function [47,49] in comparison to appropriate control groups aimed at disentangling the specific contribution of motor and non-motor components of the training activities. Moreover, other studies explored the effects of meditative practices on creativity adopting the AUT [30,54]. However, so far previous research on sitting and movement meditative practices examined training-induced effects on these two tasks mostly in adults. In order to extend from adulthood to childhood research, we ensured consistency in testing to clearly evaluate the generalizability from adulthood to childhood.

Moreover, the choice to specifically link a divergent thinking (AUT) assessment of creativity and a spatial cognition (HFT) assessment is justified by the fact that they both rely on visual perception, representation and imagery of objects. We stress that divergent thinking tests are estimates of the potential for creative problem solving rather than overall creativity tests [74], in the case of a visuospatial divergent thinking test as the AUT spatial cognition is inherently linked to the ability to see a visuospatial problem from different perspectives and find more divergent solutions [57].

For the HFT, the children were asked to identify a simple geometrical figure embedded in a complex figure in order to evaluate visuospatial abilities and field independency [75,76]. Children were administered eight different figures at each assessment and had a time limit of 30 s to complete the task for each figure. The order of figures was randomized between participants. A different version of the test was used for each assessment. The outcome measure was the number of correct identifications of figures (“hits”).

For the AUT, the children were asked to write as many different uses as they could imagine for each target object in order to provide measurements of creativity associated with cognitive flexibility [16,77,78]. Children were administered three different target objects for each assessment and had a time limit of two minutes for each target word. The triplets of objects presented in each test session were counterbalanced across classes and assessments. In this way, every target object was presented only once for each child and for only one class in each assessment, thus avoiding possible repetition effects. Additionally, counterbalancing allowed to avoid the systematic coupling of a given object with a given experimental condition and therefore the risk that differences in alternative uses found for different objects could co-vary with the experimental conditions of interest. The target objects utilized in this study were selected from a larger database according to their scores in concreteness, contextual availability, and familiarity (for target selection details, [48]). Two independent raters manually scored the AUT. After a first round of scoring, inter-rater agreement was 87.8% (330 disagreements on 2701 total responses). Divergent scorings were discussed until the raters reached 100% agreement. For data analysis, we extracted three indexes: Fluency, Flexibility, and Elaboration [79]. Fluency was the total number of uses mentioned for each object. Flexibility was the total number of categories to which each use could be ascribed (e.g., regular use, metaphorical use, weapon, etc.). Elaboration reflected the amount of additional information that enriched the mentioned use (e.g., contextual information, other actors involved, etc.).

In each session, tests were presented in the same order, starting with the AUT and then administering the HFT. An invariant order of testing (see, e.g., [80]) has been described as being “standard practice” [81], and has been justified as serving “to avoid confounding order effects with individual differences” [82].

A group simulation of both tests was performed before each assessment to ensure that all children correctly understood the task instructions.

### 2.5. Statistical Analysis

Outcome measures of our study were: (1) the effect of the two types of interventions on cognitive functions relevant for school learning, and (2) the maintenance/decay of any training effects during the washout period to inform implementation in future studies.

To assess the efficacy of the interventions, delta values related to the changes occurred between the first two assessments in the first training phase (t1–t0) and between the first two assessments in the second training phase (t4–t3) were computed for the correct number of identified figures in the HFT and the three indices of creativity in the AUT. Thus, for the HFT analysis, the dependent variable was the change in the number of identified figures (ΔHits), while for the AUT analysis, variables of interests were the changes in Fluency, Flexibility, and Elaboration.

Then, in order to compare the effects of the two trainings also as a function of age (school grade), we entered the delta scores in separate ANOVAs, each having Training (QMT vs OMM) as a within-participants factor and Class (5th vs 6th vs 7th vs 8th grade) as a between-participants factor. In the case of a main effect for Class or a significant Training x Class interaction, delta values were submitted to planned comparisons (*t*-tests) with Bonferroni correction for multiple comparisons (*p* < 0.006 for eight comparisons). Effect sizes (Cohen’s d; [83]) and 95% CI were computed separately for each training type and school grade (for delta values ≠ 0) regardless of the significance of the Training x Class interaction, in order to provide effect sizes for future sitting/movement meditation interventions with children of different ages.

For the second outcome measure, we compared the baselines of the two training phases (t0 and t3) using a paired-sample *t*-test for each index of spatial cognition and creativity separately for each school grade (class) in order to assess whether performance after the washout returned to the levels of the first assessment. Alpha values were adjusted with Bonferroni correction for the number of pairwise comparisons (*p* < 0.0125 for four comparisons). Effect sizes (Cohen’s d) with 95% confidence interval (CI) were also computed.

All analyses were conducted with IBM SPSS (IBM corp., IBM SPSS for Windows, Version 26.0, Armonk, NY, USA), except for effect size computations that were performed using online tools [84,85].

Since data collection was stopped due to the COVID-19 lockdown in the middle of the last training phase, we decided to focus our analysis on available comparable training periods (i.e., t0-to-t1 and t3-to-t4) excluding the data collected at the end of the second half of the first training phase (i.e., at t2).

### 2.6. Ethical Statement

This study was approved by the ethical committee of Bar-Ilan University in Israel.

## 3. Results

### 3.1. Effects of QMT/OMM Trainings and Moderation by Age

#### 3.1.1. HFT

A significant main effect of Class [*F*(3,35) = 3.41, *p* < 0.05] and a significant Training x Class interaction [*F*(3,35) = 3.41, *p* < 0.05] without a significant main effect of Training (*p* = 0.75) were observed. Bonferroni corrected pairwise comparisons showed a statistically significant difference between trainings for the 5th graders (*p* < 0.05), suggesting that 5th graders performed better following QMT compared to OMM. The 6th graders showed a significant difference between trainings as well, but in reverse, with 6th graders performing better following OMM compared to QMT (*p* < 0.05). The differences between trainings among the 7th and 8th graders were not significant (both *p* > 0.79), suggesting that QMT and OMM had a similar effect on task performance.

**Figure 3:**
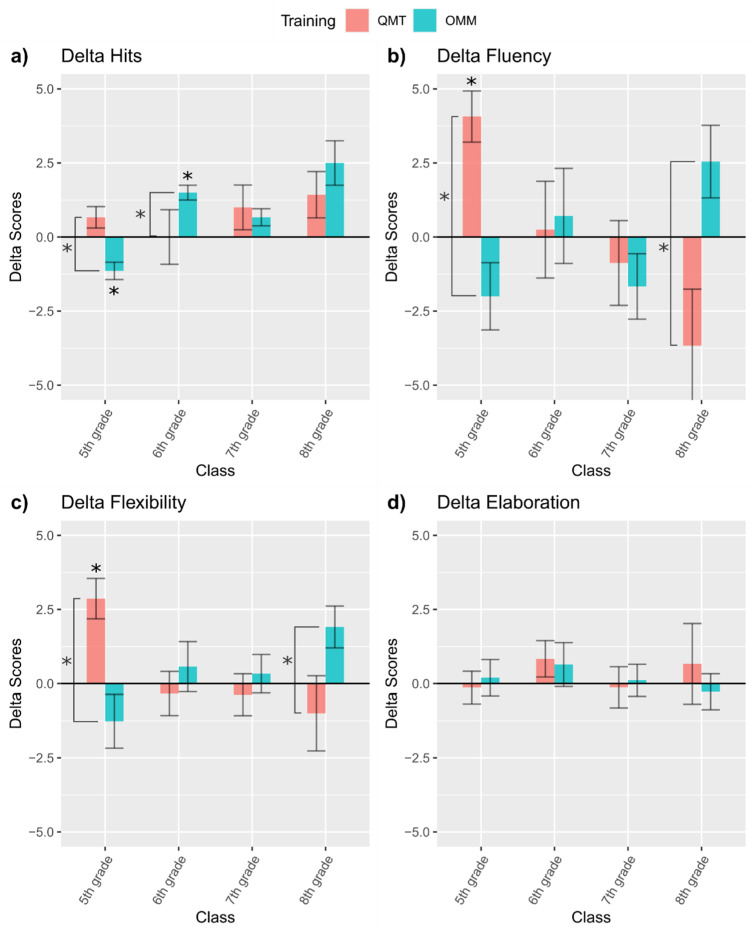
Delta scores for (**a**) Hits in Hidden Figure Test (HFT); (**b**) Fluency in Alternative Uses Test (AUT); (**c**) Flexibility in AUT; (**d**) Elaboration in AUT. Significance levels reported above bars mean that the score is significantly different from 0 with Bonferroni corrected alpha: * = *p* < 0.006; significance levels reported on the side of bars mean that ANOVA revealed significantly different scores between trainings: * *p* < 0.05.

One-sample *t*-test analysis on ΔHits showed no significant improvements in HFT performance following QMT among all grades (*p*-values > 0.08). In contrast, following OMM, HFT performance showed a significant reduction among 5th graders (*p*-value < 0.006) and a significant improvement among 6th graders (*p* < 0.006). No significant changes in 7th and 8th graders resulted significant following Bonferroni correction (*p*-value > 0.006) (Figure 3a).

Effect sizes were moderate for 5th, 7th, and 8th graders following QMT, and moderate to large for all grades following OMM (see Table 1).

Overall, no significant effects were found in ΔHits following QMT even if we observed moderate effect size; instead, we observed significant effects in 5th and 6th graders and moderate to large effect sizes in all grades following OMM.

#### 3.1.2. AUT

##### Fluency

No significant main effects of Training or Class were observed (*p* > 0.39). However, there was a significant Training x Class interaction [*F*(3,36) = 7.11, *p* < 0.001]. Bonferroni corrected pairwise comparisons showed a statistically significant difference between trainings in 5th graders (*p* < 0.001), suggesting that 5th graders had better fluency following QMT compared to OMM. The 8th graders showed a significant difference between trainings as well, but a reversed finding, with 8th graders having better fluency following OMM compared to QMT (*p* < 0.05). The differences between trainings in 6th and 7th graders were not significant (both *p* > 0.53), suggesting that QMT and OMM had a similar effect on fluency.

One-sample *t*-test analysis on ΔFluency showed significantly improved fluency among 5th graders following QMT (*p* < 0.001), while all other grades showed no QMT-related improvements (*p* > 0.11). No significant improvements in fluency were observed following OMM (all *p* > 0.06) (Figure 3b).

Following QMT, effect sizes were large in 5th graders, moderate in 8th graders, small in 7th graders, and no effect was observed in 6th graders. Following OMM, no effect was observed in 6th graders, while moderate effect sizes were found for all other grades (see Table 2). Overall, younger children (5th grade) showed significantly improved fluency with a large effect size following QMT, and no significant improvements with small to moderate effect sizes following OMM.

##### Flexibility

No significant main effects of Training or Class were observed (*p* > 0.68). However, there was a significant Training x Class interaction [*F*(3,36) = 7.08, *p* < 0.001]. Bonferroni corrected pairwise comparisons showed a statistically significant difference between trainings among 5th graders (*p* < 0.001), suggesting that 5th graders had better flexibility following QMT compared to OMM. The 8th graders showed a significant difference between trainings as well, but a reversed finding, suggesting that 8th graders had better flexibility following OMM compared to QMT (*p* < 0.05). The differences between trainings in 6th and 7th graders were not significant (both *p* > 0.51), suggesting that QMT and OMM had a similar effect on flexibility.

One-sample *t*-test analysis on ΔFlexibility showed a significant improvement in flexibility following QMT only in 5th graders (*p* < 0.001), but not in all other grades (*p* > 0.46). No significant improvement in flexibility following OMM was observed, except for a positive change in 8th graders that resulted non-significance after applying the Bonferroni correction (*p* > 0.006) (Figure 3c).

Following QMT, the effect size was large in 5th graders and null to small in all other grades. Following OMM, the effect size was large in 8th graders and null to small for all other grades (Table 3). Overall, younger children (5th grade) showed significantly improved flexibility with a large effect size following QMT. No significant improvements and small effect sizes were observed following OMM, except for a large effect size in the case of 8th graders.

##### Elaboration

No significant main effect or interaction was observed (all *p*-values > 0.56). Since no main effect of Training or Training x Class was significant, one-sample *t*-test analysis was not computed for Elaboration (Figure 3d).

We observed null effect sizes in 5th and 7th grades and null to small effect sizes in 6th and 8th grade following both QMT and OMM (see Table 4).

### 3.2. Baseline Differences before and after the Wash-Out Period

#### 3.2.1. HFT

**Figure 4:**
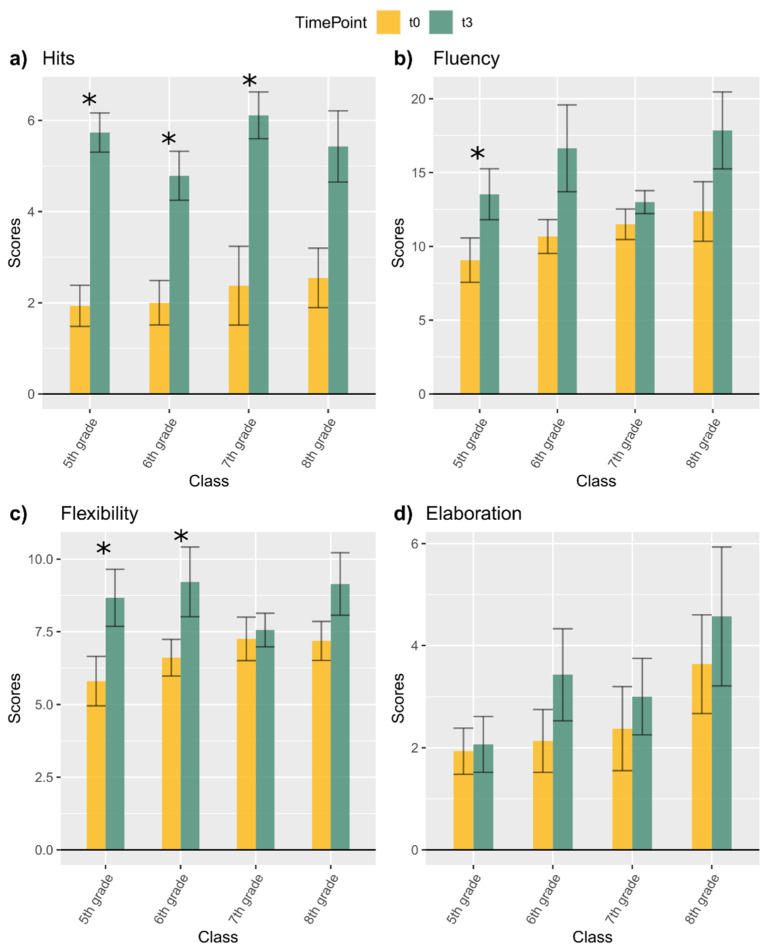
Comparisons between the baseline of the first training (t0) and the baseline before the second training (t3) for (**a**) Hits in HFT; (**b**) Fluency in AUT; (**c**) Flexibility in AUT; (**d**) Elaboration in AUT. Significance levels: * = *p* < 0.0125 (Bonferroni corrected alpha).

The amount of hits at the second baseline were significantly higher than the hits at the first baseline for the 5th, 6th, and 7th graders (all *p*-values < 0.0125), while for the 8th graders, this difference did not reach the adjusted α level (*p* = 0.035) (Figure 4a). Cohen’s *d* revealed a large effect size for all classes (5th grade *d* = 2.22, CI = 0.93–3.51; 6th grade *d* = 1.49, CI = 0.29–2.56; 7th grade *d* = 1.89, CI = 0.27–3.38; 8th grade *d* = 1.36, CI = −0.08–2.81). In general, we observed that, in the assessment immediately after the washout (t3), children’s performance was better than in the first baseline assessment (t0).

#### 3.2.2. AUT

##### Fluency

Fluency was significantly greater at the second than the first baseline only for 5th graders (*p* < 0.0125), but not for children in higher grades (Figure 4b). Cohen’s *d* revealed a moderate effect size for all classes except the 8th graders, who showed a large effect size (5th grade *d* = 0.71, CI = −0.33–1.76; 6th grade *d* = 0.71, CI = −0.33–1.75; 7th grade *d* = 0.56, CI = −0.76–1.89; 8th grade *d* = 0.81, CI = −0.55–2.16). In general, in the assessment immediately after the washout (t3), children’s fluency was overall better than in the first baseline assessment (t0) but this difference was significant only for the 5th graders.

##### Flexibility

Flexibility was significantly greater at the second than the first baseline for 5th and 6th graders (*p*-values < 0.0125), but not for children in higher grades (Figure 4c). Cohen’s *d* revealed a large effect size for 5th graders, moderate effect size for 6th and 8th graders, and a small effect size for 7th graders (5th grade *d* = 0.81, CI = −0.24–1.86; 6th grade *d* = 0.72, CI = −0.32–1.76; 7th grade *d* = 0.15, CI = −1.15–1.46; 8th grade *d* = 0.76, CI = −0.58–2.12). In general, in the assessment immediately after the washout (t3), children’s flexibility was better than in the first assessment (t0) for the 5th and 6th graders, while this difference was not significant for the 7th and 8th graders.

##### Elaboration

Elaboration was not significantly different between the first and second baseline in any of the classes involved (all *p*-values > 0.25) (Figure 4d). Cohen’s *d* revealed a small effect size for the 5th graders and moderate effect sizes for the 6th, 7th, and 8th graders (5th grade *d* = 0.06, CI = −0.94–1.08; 6th grade *d* = 0.44, CI = −0.58–1.46; 7th grade *d* = 0.27, CI = −1.04–1.58; 8th grade *d* = 0.27, CI = −1.03–1.58). In general, the children’s elaboration was not different between first (t0) and second (t3) baseline assessment.

## 4. Discussion

The current research focused on two school-based health promotion training programs for elementary and middle schoolers: movement meditation (QMT) and sitting meditation (OMM). More specifically, it was aimed to assess the feasibility and the potential outcomes of an intervention program organized as a part of the standard didactic activities in an elementary and middle school. Previous studies suggested that short activities integrated in the curricular school time could promote cognitive, emotional, and social well-being [4,43].

The rationale to perform this comparison of a sitting versus a movement-based meditation can be summed up in four points:

1. Motor activity has been linked to wellbeing and development of cognitive functions in children [4];
2. Among different types of motor activities, cognitively engaging and mindful movements have provided the most consistent evidence of positive impact on high-level cognition [86,87];
3. The implementation of short cognitively challenging motor activities in schools seems both a feasible and efficacious strategy for supporting physical and psychological well-being [43];
4. As QMT has been observed to induce neuroplasticity in adults [46], children, who have still not reached the zone of optimal cognitive functioning, could obtain greater benefit from its practice.

The preliminary findings of the current study showed that 5 weeks of daily motor and non-motor training could respectively improve children’s cognition. More specifically, younger children showed better spatial cognition and greater fluency and flexibility following the movement-based meditation (QMT), while older children showed greater creativity following the sitting meditation training (OMM) and greater visuospatial abilities following both trainings. These findings suggest that the two trainings employed in the current study could differently affect cognition accordingly to the children’s developmental stage.

As the subjective experience of the world is, first of all, a physical experience of the spatial world [88,89], several models of consciousness and, consequently, of cognition, have emphasized the importance of space and its representations [19,90,91]. Spatial representations are thought to serve as the base for embodied cognition, underlie abstract thought and rely on similar brain mechanisms [92,93]. Based on this, the Sphere Model of Consciousness (SMC) suggests that through body-centered meditative practices, such as QMT and OMM [58,59] one can detach from his/her habitual relationship with space, and consequently detach from the habitual self and its ways of thinking.

QMT is a movement-based meditation; as such, its practice activates and focuses attention to the bodily self through engagement of the motor system. Importantly, the execution of movement in space promotes the generation of external and internal space mappings [94]. The activation of the bodily self and the contextual generation of internal and external spatial representations both provide a sense of being in the here-and-now and promote visuospatial abilities [47,73].

Our results suggest that the motor component of QMT exerts a pervasive effect in promoting development in different cognitive domains in children as young as 10 years through generation of a correspondence between internal and external space, with improvements of moderate to large effect sizes in visuospatial abilities (*d* = 0.48) and in two creativity indexes (*d* = 1.21 for fluency; *d* = 1.08 for flexibility). Additionally, QMT improved visuospatial abilities in older children too (13 year-old children, *d* = 0.69), suggesting that the motor component of movement-based meditation could influence the developmental trajectory of specific spatial-related cognitive functions during a longer period of the developmental window than that in which effects on creativity could be observed.

The OMM, on the other hand, is a sitting meditation technique in which practitioners are trained to split their attention between their current bodily state and the formation of their ‘best self’ through self-awareness of their own desires, emotions, and thoughts. As OMM does not involve bodily movement, the generation of internal spatial representation could be putatively related to imagery mechanisms. In our study, following OMM, children in the 6th, 7th, and 8th grade showed improvements of visuospatial abilities with moderate to large effect sizes (*d* = 1.59, 0.77, 1.05, respectively), while younger children showed reduced visuospatial performance with a large effect size (*d* = −1.04).

Noteworthily, the fact that 5th graders showed significant improvement of HFT performance only after QMT and 6th graders only after OMM may be at least partially due to a learning effect of the testing task, since the QMT and OMM represented the first training for 5th and 6th graders, respectively. This interpretation is, however, less likely, because a similar pattern of learning effect due to the sequence of trainings should have been observed in the same way for 7th and 8th graders. This information should be used for the development of future studies to rule out the possibility that differential effects of the QMT/OMM trainings are attributable to a design-related effect.

Our hypothesis on the involvement of motor imagery in the generation of internal spatial representations is supported by the observation that 12-year-old children show better motor imagery abilities compared to younger children, highlighting their ability to manipulate motor and spatial representations in the absence of overt movements [95]. Thus, OMM, which lacks overt movement but stimulates the generation of internal space through imagery, may improve visuospatial abilities of older children, who in contrast to younger children, can capitalize on motor imagery to improve visuospatial abilities.

Executive functions underlying perceptuomotor learning are already developed enough at approximatively 10 years old, and that corresponds to the age of the youngest group of participants in our study (5th grade) [96]. At the same age, gray matter reaches its maximal thickness and then decreases contextually with an increases of white-matter and cerebellar volume that continue to develop into adolescence until adulthood [97,98]. This trade-off between grey and white matter development has been associated with age-related differential outcomes in visuo-motor sequences learning, showing a positive correlation between age and measures of effective sequence learning (i.e., accuracy and timing) [99].

Considering these developmental phenomena, we hypothesize that 10-year-old children have the set of cognitive functions necessary to successfully learn and perform meditative sensorimotor trainings, such as QMT. Nevertheless, the performance could be more challenging for younger compared to the older group, thus resulting in greater engagement leading to greater efficiency in promoting cognitive improvements in the younger group.

Considering that 5th grade is part of elementary school while all the other classes involved in this study are part of the middle school, we can also hypothesize that developmental processes together with a different didactic setting could have affected the development of motor imagery, producing different outcomes in visuospatial abilities. Further studies on the effect of didactic settings on visuospatial abilities and imagery are necessary.

Contrary to what was observed in younger children, older children showed improvements with moderate to large effect sizes in two indexes of divergent thinking exclusively following OMM training (*d* = 0.62 for fluency; *d* = 0.81 for flexibility), suggesting that this kind of sitting meditation is better suited to foster creativity in 13 year-old children. The effect of meditation on creativity in children has already been predicted [100]; however, we are unaware of any study that directly explored this relationship. Nevertheless, our preliminary results are in line with other studies showing that meditative practices could increase divergent thinking and creativity in adults [30,54] and young adults [101].

With regards to the relationship between OMM and creativity, we suggest that older children obtained greater benefits compared to younger ones because they have more developed abstract thinking which is closely linked to creativity [102]. Our interpretation is that OMM promotes abstract thinking development by allowing greater coupling between an ‘abstract’ narration of the self with the experience of the bodily self, providing a mechanism that promotes embodiment of thoughts referring to the narrative there-and-then in the bodily here-and-now.

Furthermore, in relation to creativity and memory, it’s important to note that short-term memory is connected to the Minimal Self [68], which has a shorter temporal extension with respect to the narrative self. In addition, short-term memory is important to wider attention. For example, when one is in a state of flow, one can bring attention to multiple things without forgetting.

## 5. Future Directions and Limitations

In order to better integrate these trainings within the curricular school time, we designed the present crossover study in a way that the training phases were performed contextually within the established scholastic periods (i.e., from September to December for the first phase and from January to April for the second phase) with the washout overlapping with the winter holiday break. However, our results revealed that the washout was not long enough to abate the effect of the first training phase. While this is a positive indicator of the effect’s maintenance over time, task learning and developmental processes could also have affected the second baseline measurement. In fact, strong learning effects related to the HFT have been observed in the literature [103]. To rule out a learning effect and other confounding developmental processes, future studies should include a passive control group. Moreover, considering that the current study included one elementary school class and three middle-school classes, the difference in teaching strategies and settings between classes children were clustered in could have acted as a confounding variable. Future studies should consider including a larger amount of classes from the same setting and taking into account the clustered nature of the data for the definition of the sample size needed to achieve adequate power. The effect sizes found in this study as a function of meditation type and age are useful for this aim.

Regarding a methodological aspect of future research, we advise that other tests could be adopted in order to provide a clearer picture of the cognitive and behavioral changes that could be affected by the trainings. Especially the study of creativity that is known to be a multifaceted construct [104,105], other tests exploring not only other dimensions of divergent thinking but also convergent thinking, figural and motor creativity could be taken into consideration.

Finally, additional control groups could be taken into consideration, such as physical and nonphysical exergames and other group-based interventions [106,107].

## 6. Conclusions

The current study showed that an integrative approach aimed at enriching didactic programs with active meditative sessions could provide potential benefit in cognitive development. In light of what we observed, we recommend that future studies: (1) implement shorter training phases in order to assess how the intervention time may affect the maintenance of the effects after training cessation in an ecologically valid time context (e.g., established scholastic holiday breaks); (2) include a passive control group to rule out the effect of training-unrelated cognitive development; and (3) let the children familiarize with the tests before the first assessment.

The results of the present pilot study, despite being preliminary, are interesting. The choice of the training methodology for future studies should take in consideration the developmental stage of the children, since we observed different effects across classes and trainings. The observation of moderate to large effect sizes for at least two grades utilizing different trainings encourages the design of future well-powered trials aimed at assessing the effects of movement-based and sitting meditations in children.

Note: Note that the factor Class covaried with the sequence of administration of the two training types, with 5th and 7th graders starting with QMT and 6th and 8th graders with OMM (Figure 2). Thus, the better HFT performance following QMT in the case of 5th graders but following OMM in the case of 6th graders might be attributable to a learning effect that has been reported in the literature for the HFT test [103]. In order to explore this potential learning effect, we collapsed the HFT scores across trainings and classes and performed a repeated measures ANOVA to compare the HFT performance at adjacent time-points (t0–t1, t1–t2, t2–t3, t3–t4). This analysis showed progressive, significant increases from t0 to t2 through t1, followed by a plateau across the following time points.

## Figures and Tables

**Table 1:** Effect size (Cohen’s d) and 95% confidence intervals for ΔHits following QMT (left) and OMM (right) in each class.

	QMT			OMM		
	Cohen’s d	Lower CI	Upper CI	Cohen’s d	Lower CI	Upper CI
5th grade	0.48	−0.06	1.01	−1.04	−1.66	−0.39
6th grade	0	\	\	1.59	0.81	2.35
7th grade	0.47	−0.23	1.14	0.77	0.01	1.51
8th grade	0.69	0.01	1.33	1.05	0.29	1.79

Note: since no changes were observed following QMT in 6th graders (ΔHits = 0), effect size and confidence intervals were not computed. QMT = Quadrato Motor Training; OMM = One Minute Meditation; CI = 95% Confidence Intervals.

**Table 2:** Effect size (Cohen’s d) and 95% confidence intervals for ΔFluency following QMT (left) and OMM (right) in each class.

	QMT			OMM		
	Cohen’s *d*	Lower CI	Upper CI	Cohen’s *d*	Lower CI	Upper CI
5th grade	1.21	0.53	1.88	−0.45	−0.98	0.08
6th grade	0.04	−0.46	0.54	0.11	−0.39	0.62
7th grade	−0.21	−0.87	0.45	−0.50	−1.18	0.21
8th grade	−0.78	−1.45	−0.08	0.62	−0.03	1.26

**Table 3:** Effect size (Cohen’s d) and 95% confidence intervals for ΔFlexibility following QMT (left) and OMM (right) in each class.

	QMT			OMM		
	Cohen’s *d*	Lower CI	Upper CI	Cohen’s *d*	Lower CI	Upper CI
5th grade	1.08	0.43	1.71	−0.36	−0.87	0.16
6th grade	−0.13	−0.63	0.38	0.18	−0.33	0.68
7th grade	−0.19	−0.85	0.46	0.17	−0.49	0.82
8th grade	−0.32	−0.92	0.29	0.81	0.11	1.49

**Table 4:** Effect size (Cohen’s d) and 95% confidence intervals for ΔElaboration following QMT (left) and OMM (right) in each class.

	QMT			OMM		
	Cohen’s *d*	Lower CI	Upper CI	Cohen’s *d*	Lower CI	Upper CI
5th grade	−0.06	−0.56	0.44	0.08	−0.42	0.59
6th grade	0.39	−0.14	0.91	0.23	−0.28	0.74
7th grade	−0.06	−0.71	0.58	0.06	−0.58	0.72
8th grade	0.20	−0.40	0.79	−0.13	−0.72	0.46

## Data Availability

The data presented in this study are available on request from the corresponding author. The data are not publicly available due to privacy policy.
